# Immune landscape-based machine-learning–assisted subclassification, prognosis, and immunotherapy prediction for glioblastoma

**DOI:** 10.3389/fimmu.2022.1027631

**Published:** 2022-12-01

**Authors:** Haiyan Li, Jian He, Menglong Li, Kun Li, Xuemei Pu, Yanzhi Guo

**Affiliations:** College of Chemistry, Sichuan University, Chengdu, Sichuan, China

**Keywords:** glioblastoma (GBM), immune landscape, machine-learning (ML), subclassification, prognosis, immunotherapy

## Abstract

**Introduction:**

As a malignant brain tumor, glioblastoma (GBM) is characterized by intratumor heterogeneity, a worse prognosis, and highly invasive, lethal, and refractory natures. Immunotherapy has been becoming a promising strategy to treat diverse cancers. It has been known that there are highly heterogeneous immunosuppressive microenvironments among different GBM molecular subtypes that mainly include classical (CL), mesenchymal (MES), and proneural (PN), respectively. Therefore, an in-depth understanding of immune landscapes among them is essential for identifying novel immune markers of GBM.

**Methods and results:**

In the present study, based on collecting the largest number of 109 immune signatures, we aim to achieve a precise diagnosis, prognosis, and immunotherapy prediction for GBM by performing a comprehensive immunogenomic analysis. Firstly, machine-learning (ML) methods were proposed to evaluate the diagnostic values of these immune signatures, and the optimal classifier was constructed for accurate recognition of three GBM subtypes with robust and promising performance. The prognostic values of these signatures were then confirmed, and a risk score was established to divide all GBM patients into high-, medium-, and low-risk groups with a high predictive accuracy for overall survival (OS). Therefore, complete differential analysis across GBM subtypes was performed in terms of the immune characteristics along with clinicopathological and molecular features, which indicates that MES shows much higher immune heterogeneity compared to CL and PN but has significantly better immunotherapy responses, although MES patients may have an immunosuppressive microenvironment and be more proinflammatory and invasive. Finally, the MES subtype is proved to be more sensitive to 17-AAG, docetaxel, and erlotinib using drug sensitivity analysis and three compounds of AS-703026, PD-0325901, and MEK1-2-inhibitor might be potential therapeutic agents.

**Conclusion:**

Overall, the findings of this research could help enhance our understanding of the tumor immune microenvironment and provide new insights for improving the prognosis and immunotherapy of GBM patients.

## Introduction

Glioblastoma (GBM) is the most common malignant primary brain tumor, accounting for 82% of all malignant gliomas (1). Due to its malignant growth and invasion into the brain parenchyma, coupled with resistance to chemotherapy and targeted therapy, GBM is the deadliest cancer among all cancers (2). GBM could be divided into four subtypes based on an unsupervised gene expression analysis by Verhaak et al. in 2010, including classical (CL), mesenchymal (MES), proneural (PN), and neural (NE), each featuring distinct genetic, epigenetic, and transcriptional alterations (3). CL GBM has a high rate of EGFR gene amplification and expresses the markers of neuron precursor cells and stem cells (4, 5). MES reveals the features of cultured astrocytic gliomas with predominant NF1 gene aberrations and PTEN mutations. It is commonly linked to a poor prognostic outcome (4, 6) and also shows the highest inflammatory signature with significant upregulation of genes in the TNF and NF-κB pathways (7). PN is characterized by a lower incidence rate and the best median patient survival. It has PDGFRA alterations and point mutations of IDH1 and develops mainly in younger patients with secondary glioblastoma (5, 7). Clustered in the normal brain samples, NE shows strong expression of neuronal markers including NEEL, GABRA1, SYT1, and SLC12A5 (6). So, we can see that different GBM subtypes exhibit a high degree of inter- and intratumor heterogeneity.

Immunotherapy, represented by immune checkpoint blockage (ICB), has been becoming an appealing treatment for gliomas. Immune checkpoint inhibitors (ICIs) can induce an improved clinical response in patients, and emerging evidence has disclosed that the anticancer efficacy of ICIs is dependent on the tumor microenvironment (TME) (8). As a vital mediator of tumor malignant progression and therapeutic outcome, TME is closely associated with the immune evasion of tumor cells. The GBM microenvironment mainly consists of non-neoplastic cells, infiltrating and resident immune cells, vascular cells, and other glial cells (9). In the TME, through infiltration into tumor tissue to form the tumor immune microenvironment (TIME), immune cells could help tumor cells achieve immune escape and promote tumor malignancy, which is closely associated with the response rate of immunotherapy (10). It is known that there is a highly heterogeneous immunosuppressive microenvironment in GBM (11–13); therefore, it is of great practical significance to explore the differences in immune landscapes among GBM subtypes.

Recently, almost all studies focusing on the GBM immune landscape selected only one of the different immune signatures to divide GBM patients into different immune phenotypes, such as immune-related genes (1, 14), lncRNAs (15), immune cell infiltration-associated genes (16), abundances of immune cells (10, 17), and antigen presentation machinery (APM) signature (18). Moreover, for the immune landscape of GBM subtypes, Doucette et al. have studied the associations of antigen expression, immunosuppression, and effector response genes within GBM subtypes (19). The distribution and the infiltration of the immune components across the commonly described subgroups have been analyzed by Maria et al. using an immunohistochemistry-based approach (6). Until now, there has been no comprehensive analysis of the immune landscape among GBM subtypes that integrates various immune characteristics.

Meanwhile, machine learning (ML)-based methods could detect key features from complex datasets and have been popular applications in clinical cancer research in recent years, such as early diagnosis, subtype identification, prognosis prediction, and so on (20). It has been used to classify various cancer subtypes, for example, breast cancer (21), adult T-cell leukemia/lymphoma (22), kidney cancer (23), and glioma (24). The application of ML for cancer subtype identification will enable accurate diagnosis and regard to the clinical management of patients. By performing a comprehensive immunogenomic analysis, we aim to develop ML-based models to achieve a precise diagnosis, prognosis, and immunotherapy prediction for GBM.

In this study, we integrated the expression profiles of 397 GBM samples from public databases and studies. Single-sample gene-set enrichment analysis (ssGSEA), xCell, ESTIMATE, and other algorithms were employed to collect the largest number of immune signatures, and 109 immune signatures were utilized to comprehensively characterize the immune landscapes of GBM subtypes.

Initially, we focused on constructing an immune signature-based model for the accurate recognition of different GBM subtypes using ML methods. Among 109 features, 61 were proved to yield great contributions to the identification of three GBM subtypes, and the RBF-based support vector machine (SVM) gives the best diagnostic performance. Moreover, the prognostic values of these 61 optimal immune features were determined, and a prognostic risk model was established for the OS prediction of GBM patients based on 13 survival-associated immune signatures using multivariate Cox regression analysis. A complete differential analysis across GBM subtypes was then performed on the optimal immune characteristics. Moreover, clinicopathological and molecular features were also considered for comparisons of the three subtypes. Analyses of exhausted CD8+T cells and anti-PD-1 immunotherapy response were also conducted. Eventually, based on the differential upregulated genes of MES compared to CL and PN samples, gene-set cancer analysis (GSCA) and connectivity map (CMap) analysis were also performed to achieve potential antitumor drugs or small molecules for MES patients.

## Materials and methods

### Data collection and preprocessing

GBM samples are commonly classified into four subtypes CL, MES, PN, and neutral (NE), respectively. Currently, only a few NE samples are available, so we mainly considered the three classes of CL, MES, and PN. We downloaded three gene expression profiling datasets along with the corresponding clinical information for the three subtypes, including TCGA-GBM from the TCGA data portal (https://portal.gdc.cancer.gov/), the Gravendeel microarray dataset from the GlioVis database (http://gliovis.bioinfo.cnio.es/), and the Wang RNA-seq dataset (25), respectively. After deleting samples without either expression data or clinical information, 397 eligible GBM samples remained, consisting of 131 CL, 140 MES, and 126 PN samples. Firstly, the missing gene expression data in the Wang RNA-seq dataset were complemented by the K-nearest neighbor (KNN) method. These gene expression data were then transformed into transcripts per kilobase million (TPM) format so as to calculate immune characteristics. Finally, the ComBat method from the “SVA” R package was used to remove the batch effects among three different datasets.

### Immune landscape construction

Here, we aim to collect the largest number of immune characteristics to construct comprehensive immune landscapes for GBM samples. By performing a deep exploration of the literature, various immune characteristics were acquired, including 28 tumor-infiltrating lymphocytes, 64 immune and stromal cells, the cytolytic score (CYT score), the T-cell infiltration score (TIS score), the immune score, the stromal score, tumor purity, the innate and adaptive immune scores, the immune checkpoint gene score (ICG score), the APM score, the T-cell exhaustion markers, and the corresponding genes of glioma antigens.

Firstly, the ssGSEA algorithm was employed to quantify the relative abundances of 28 tumor-infiltrating lymphocytes, the TIS score, the innate and adaptive immune scores, and the APM score. The gene sets for 28 tumor-infiltrating lymphocytes were obtained from the TISIDB database (http://cis.hku.hk/TISIDB/), and those for calculating the TIS score were from the studies of Şenbabaoğlu et al. (26). From the work of Charoentong et al. (27), a set of genes that mark each infiltrating immune cell type were obtained for innate and adaptive immune scores. These gene sets are shown in **Supplementary Tables S1–S3**. For the APM score, the following genes were collected for estimation of the APM signature: PSMB5, PSMB6, PSMB7, PSMB8, PSMB9, PSMB10, TAP1, TAP2, ERAP1, ERAP2, CANX, CALR, PDIA3, TAPBP, B2M, HLA-A, HLA-B, and HLA-C (18).

The abundances of 64 various cell types were then achieved using the “xCell” R package (28). The CYT score, representing cytolytic activity, was calculated as the geometric mean of two genes’ expression, including GZMA and PRF1 using the established methodology by Takahashi et al. (8). The ESTIMATE algorithm was used to evaluate the immune score, stromal score, and tumor purity of each GBM sample for determining the immune infiltration level in the tumor. We computed the average expression value of six immune checkpoint genes, including PDCD1, CD274, CTLA4, HAVCR2, LAG3, and TIGIT, as the ICG score of every GBM sample.

Moreover, we also assessed the expressions of T-cell exhaustion markers and corresponding genes of glioma antigens (6). They are composed of 11 genes (PDCD1, CD274, CTLA4, IDO1, IDO2, LAG3, HAVCR2, PDCD1LG2, TIGIT, ADORA2A, and VTCN1) and 17 genes (EGFR, ERBB2, BIRC5, NCL, EPHA2, TERT, CCNB1, SART1, DSE, SART3, AIM2, TYRP1, TYR, MGAT5, PMEL, MLANA, and MAGEA1), respectively.

Finally, after deleting the overlaps between 28 tumor-infiltrating lymphocytes and 64 immune and stromal cells, a total of 109 immune signatures were achieved (**Table 1**) and then normalized by z-score for further integrative immunogenomic analysis. Details can be seen in **Supplementary Table S4**.

**Table 1:** List of 109 immune signatures collected in this paper.

Feature type	Detailed signature names
Immune and stromal cells	Activated B cell, activated CD4 T cell, activated CD8 T cell, CD56bright natural killer cell, CD56dim natural killer cell, myeloid-derived suppressor cell, T follicular helper cell, Type 17 T helper cell, aDC, adipocytes, astrocytes, B cells, basophils, CD4+ memory T cells, CD4+-naive T cells, CD4+T cells, CD4+ Tcm, CD4+ Tem, CD8+-naive T cells, CD8+T cells, CD8+ Tcm, CD8+ Tem, cDC, chondrocytes, class-switched memory B cells, CLP, CMP, DC, endothelial cells, eosinophils, epithelial cells, erythrocytes, fibroblasts, GMP, hepatocytes, HSC, iDC, keratinocytes, ly endothelial cells, macrophages, macrophages M1, macrophages M2, mast cells,megakaryocytes, melanocytes, memory B cells, MEP, mesangial cells, monocytes, MPP, MSC, MV endothelial cells, myocytes, naive B cells, neurons, neutrophils, NK cells, NKT, osteoblast, pDC, pericytes, plasma cells, platelets, preadipocytes, pro-B cells, sebocytes, skeletal muscle, smooth muscle, Tgd cells, Th1 cells, Th2 cells, Tregs
Immune-related scores	CYT score, TIS score, ICG score, APM score, stromal score, immune score, tumor purity, adaptive immune score, innate immune score
T-cell exhaustion markers	PDCD1, CD274, CTLA4, IDO1, IDO2, LAG3, HAVCR2, PDCD1LG2, TIGIT, ADORA2A, VTCN1
Corresponding genes of glioma antigens	EGFR, ERBB2, BIRC5, NCL, EPHA2, TERT, CCNB1, SART1, DSE, SART3, AIM2, TYRP1, TYR, MGAT5, PMEL, MLANA, MAGEA1

### Evaluation and selection of immune features

In order to select the distinctive immune features for distinguishing three GBM subtypes, support vector machine recursive feature elimination (SVM-RFE) was used to evaluate all 109 signatures. SVM-RFE is a backward feature deletion method based on SVM, and it recursively removes the features with the lowest weights that are computed after the SVM learning model is built (29). On account of its superiority, SVM-RFE has been widely adopted for feature selection of genomics, proteomics, and metabolomics data (30). To perform a reliable and convincing feature evaluation, SVM-RFE was implemented 100 times on 109 immune signatures, and those retained more than 50 times were selected as the distinctive ones.

### ML methods for diagnostic prediction of GBM subtypes

In order to give an optimal model for diagnostic prediction of GBM subtypes, based on the selected immune signatures by SVM-RFE, the four most widely used ML methods were adopted for model construction, including support vector machine (SVM), random forest (RF), extreme gradient boosting (XGBoost), and artificial neural network (ANN), respectively.

### SVM

Proposed by Vapnik in 1992 (31), SVM is a widely used ML method in bioinformatics due to its high accuracy and ability to deal with high-dimensional data (32). As a powerful method for building a classifier, the basic idea behind it is creating a decision boundary between two classes that enables the prediction of labels from one or more feature vectors (33). It has been used successfully in various cancer identification and subtyping, including breast cancer (34), lung cancer (35), lymphoma cancer (36), adult soft tissue sarcoma (37), and others. For the SVM method, four currently available kernel functions of “linear,” “polynomial,” “RBF,” and “sigmoid” were all used to construct prediction models, respectively.

### RF

Provided by Beriman et al. in 2001 (38), RF is a classification and regression method based on the aggregation of a large number of decision trees. It is an ensemble method that grows trees as base learners and combines their predicting results by averaging; in other words, the ultimate prediction is determined by the votes of all the trees for a binary task (39, 40). RF is known for its great practical performance, particularly in high-dimensional settings, and has become a standard data analysis tool in bioinformatics (41).

### XGBoost

As a regression tree that has the decision rules as a decision tree, the XGBoost algorithm was first proposed by Chen et al. (42). It can be used for regression, classification, and ranking problems. It is a ML model that integrates multiple weak learners to achieve a stronger learning effect (43). Compared with other traditional ML algorithms, XGBoost is highly scalable and flexible. It has been shown to perform exceptionally well in a variety of tasks in bioinformatics and medicine (44).

### ANN

Inspired by the early models of sensory processing by the brain, ANN is created by stimulating a network of model neurons in a computer (45). The elementary building blocks of ANN are artificial neurons, and nodes in the neural network can be mostly divided into three layers: the input layer, the output layer, and one or more hidden layers (46). Due to the high parallelism, robustness, generalization, and noise tolerance of ANN, it has been applied in various domains, and within cancer research alone, ANN can be applied to disease diagnosis, image processing, and treatment-response forecasting (47).

### Model construction and performance evaluation

Usually, the ML methods are for binary classification. Here, a multiple-label vector was used to construct the triple-class classifier. For CL samples, the label vector is [1 0 0], MES is [0 1 0], and PN is [0 0 1]. The performance of the models developed by different ML algorithms is closely related to the hyperparameters. In order to optimize the hyperparameters of each classifier, we carried out the grid search approach and fivefold cross-validation. Finally, the model was fitted on the training set with the optimal parameters and then evaluated on the corresponding testing set. For each model, 397 GBM patients were randomly divided into training and testing sets according to the ratio of 8:2. Meanwhile, in order to prove the stability of each model, the data division was repeated 50 times, so 50 different training sets and corresponding testing sets were generated. The performance of each classification model was assessed by averaging the accuracy (ACC), Precision, Recall, and *F*1 scores from the 50 testing sets. Here, four ML methods could give seven different classifiers, and the optimal one with the best performance would be selected as the final classifier. The following are the equations of four evaluation parameters:


(1) $$ACC=\frac{TP+TN}{TP+FP+TN+FN}$$

(2) $$\mathrm{Pr}ecision=\frac{TP}{TP+FP}$$

(3) Recall=TPTP+FN

(4) F1=2∗Precision∗RecallPrecision+Recall

Where TP, FP, TN, and FN are true positive, false positive, true negative, and false negative, respectively.

### Differential analysis among GBM subtypes

To explore the differences among the three GBM subtypes, we analyzed the associations between GBM subtypes and immune, molecular, and clinical characteristics, respectively. We investigated the difference in immune signatures among three GBM subtypes using the Kruskal–Wallis test (K-W test), and a two-sided *p*< 0.05 is considered a significant difference. The Kaplan–Meier curve was calculated to detect if there were survival differences among subtypes. Moreover, the divergence of isocitrate dehydrogenase (IDH) mutation status and methylguanine methyltransferase (MGMT) promoter methylation status among the three subtypes of patients was studied. Gene-set variation analysis (GSVA) was executed by the “GSVA” package to acquire the GSVA scores of biological pathways and GO terms of each GBM patient. The “limma” package was used to investigate significantly differential pathways and GO terms between three GBM subtypes, and those with an adjusted *p*-value of< 0.05 were considered statistically significant.

### Development of prognostic model by immune features and survival analysis

A univariate Cox regression analysis was performed to analyze the relationships between immune characteristics and the OS of GBM patients. The immune features that were significantly associated with the GBM OS in the univariate Cox regression analysis were then entered into a step-wise multivariate Cox regression analysis using the “survminer” R package to select the key immune features with great prognostic values. In addition, the immune signatures with *p*-value of< 0.05 were used to build the prognostic model. The risk score of each GBM patient was calculated by the following formula:


(5) $$Risk\;score=\sum_{i=1}^{n}\beta_{i}*IF_{i}$$

Where *β_i_* is the regression coefficient and IF*_i_* is the value of the corresponding immune features. According to the cutoff value determined by X-tile, GBM patients were divided into high-, medium-, and low-risk groups. The Kaplan–Meier method and log-rank tests were implemented to estimate the differences in OS among subgroups. Furthermore, receiver operating characteristic (ROC) analysis was performed to investigate the prognosis performance of the model, and area under the ROC curve (AUC) values were calculated. Meanwhile, the stratified analysis was performed to determine whether the prognostic signature is independent of other clinical variables, including gender, age, IDH status, and MGMT status, respectively.

### Identification of gene signatures for exhausted CD8+T cells

T-cell exhaustion is a hypofunctional state characterized by the accumulation of multiple co-inhibitory checkpoint receptors consisting of PD1, CTLA4, TIM3, and LAG3 (48). We acquired the upregulated PD-1-positive gene list by selecting differentially expressed genes between PD-1-high and PD-1-negative CD8+T cells from the work of Cai et al. (49). The list incorporates 478 genes in our whole dataset (**Supplementary Table S5**). To define a gene expression signature of exhausted CD8+T cells, Pearson’s correlation analysis was implemented to assess the relationship between these upregulated genes and PDCD1. A list of genes with correlation efficiency of > 0.25 and adjusted *p*-value of< 0.05 was considered the gene signature for exhausted CD8+T cells, and the gene signature was used to conduct ssGSEA to obtain the ssGSEA score as an exhausted CD8+T cell (GET) score. Here, a higher GET score indicates the better immunotherapy response.

### SubMap analysis

To further investigate the immunotherapy responses of patients with different GBM types, SubMap analysis (50) was used to compare gene expression matrices of different subtypes with those from other cancers treated with immune checkpoint blockade therapy, including transcriptomic data from 65 patients receiving anti-PD1 therapy (51) by implementing the subclass mapping method. This step was implemented on the SubMap module of the GenePattern website (http://genepattern.broadinstitute.org/) with default parameters (num marker genes = 100, num perm = 100, and num perm Fisher = 1,000).

### Drug sensitivity prediction

We enforced the Wilcoxon test to screen out the upregulated genes of MES compared to CL and PN patients with log_2_FC of > 2 and adjusted *p*-value of< 0.05. Drug sensitivity prediction was carried out using “Drug Sensitivity of Gene Set Cancer Analysis” (GSCALite, http://bioinfo.life.hust.edu.cn/web/GSCALite/) with those upregulated hub genes as input. GSCA integrates drug sensitivity and gene expression profiling data from cancer cell lines in Genomics of Drug Sensitivity in Cancer (GDSC) and Cancer Therapeutics Response Portal (CTRP). It predicts the drug response based on the calculated correlation between mRNA expression and drug with 50% inhibitory concentration (IC_50_) (52). The negative correlations mean that the predicted drugs have potential activity.

In addition, these upregulated genes were also uploaded to the CMap online tool (https://clue.io) to predict the effect of drugs on particular gene expression patterns in tumors. The result of CMap provides a score from −100 to 100 to estimate the match between the interested genes and chemicals, and bioactive chemicals with a negative score might be candidate drugs for the treatment of patients.

## Results

### Extracting optimal immune signatures and comparisons of different ML methods

Through performing SVM-RFE 100 times on the 109 immune signatures, those that were retained with more times could probably contribute more to the identification of three GBM subtypes. There are 73 signatures retained more than 50 times in our experiment, so they were selected for comparisons of different ML methods.

**Figure 1:**
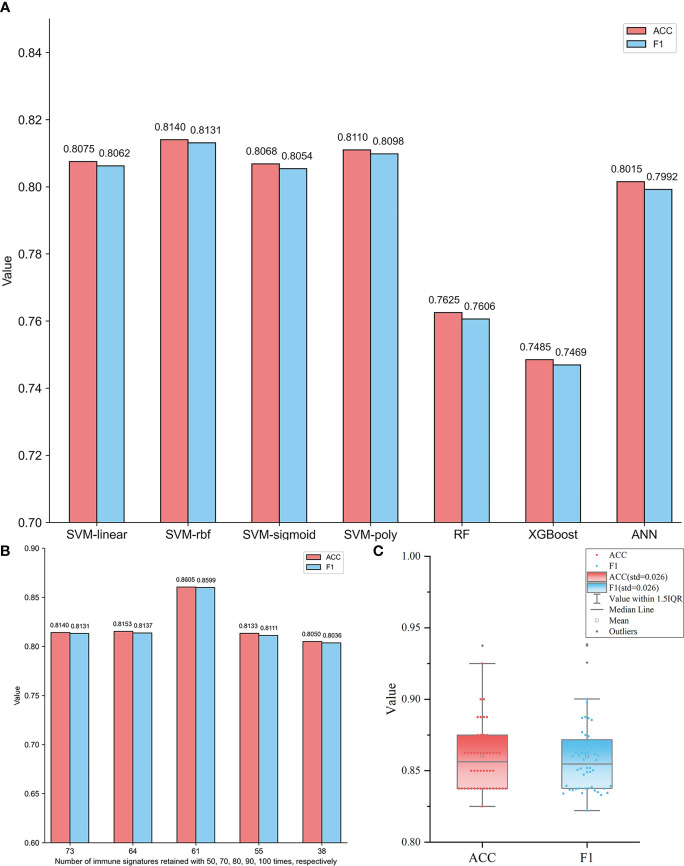
**(A)** Performance comparisons of different ML models based on 73 immune signatures retained at least 50 times in SVM-RFE 100 times. The ACC and the *F*1 values of each model are the averages of those for 50 different testing sets generated by randomly dividing each subtype dataset into training and testing sets 50 times. **(B)** Comparisons of RBF-based SVM models based on five feature subsets containing immune signatures retained at least 50, 70, 80, 90, and 100 times, respectively. The ACC and the *F*1 values of each model are the averages of those for 50 different testing sets generated by randomly dividing each subtype dataset into training and testing sets 50 times. **(C)** The distributions of ACC and *F*1 values of 50 different testing sets based on the RBF-based SVM model with 61 optimal immune signatures retained at least 80 times.

In order to give convincing comparisons, the whole dataset was divided into training and testing sets 50 times in an 8:2 ratio. Based on each training set, fivefold cross-validation was used to build the model, and the performance of the model was tested by the testing set. For seven different ML methods, the overall performance was compared by averaging those of 50 different testing sets. The comparison results are summarized in **Figure 1A**. We can see that compared to RF, XGBoost, and ANN, the SVM algorithm shows better prediction performance. Although the four different kernel function-based SVM models give comparable results, the RBF-based model exhibits slightly higher ACC and *F*1 values than the other three models. Since RBF has been the most widely used kernel for resolving nonlinear classification problems, RBF-based SVM was selected as the optimal classifying algorithm.

Using the RBF-based SVM model, the diagnostic ability of these 73 signatures was further evaluated. In order to evaluate the performance of different feature subsets for GBM subtypes identification, additional four feature subsets containing 64, 61, 55, and 38 signatures were also extracted to construct classifiers. They were retained at least 70, 80, 90, and 100 times, respectively, by SVM-RFE. **Figure 1B** shows the performance of RBF-based SVM models based on five feature subsets, respectively. The AAC and *F*1 values of each model are the averages of 50 testing sets. It is observed that in **Figure 1B**, the model based on 61 immune signatures retained at least 80 times showed significantly superior performance than others. It yields the highest ACC and *F*1 values of 0.8605 and 0.8599, respectively. So, the optimal model of RBF-based SVM with 61 signatures (**Supplementary Table S6**) was obtained. Moreover, the robustness of this model was also confirmed by the prediction results of 50 individual testing sets, which is shown in **Figure 1C**. The distributions of ACC and *F*1 values indicate that the 50 models all give a slightly varying performance, although they were constructed based on different training sets. The standard deviation (std) values are both only 0.026. So, the selected optimal model is robust.

### Construction of the final classifier

Since the best ML method of RBF-based SVM and the optimal feature subset with 61 immune signatures were selected, the final classifier was constructed using 10-fold cross-validation on the whole dataset, including 397 GBM samples. The prediction results of the final classifier are shown in **Table 2**, indicating a promising prediction performance. The ACC, Precision, Recall, and *F*1 scores in 10-fold cross-validation are 0.8538, 0.8519, 0.8621, and 0.8525, respectively. Especially, the Precision and Recall values for the MES subtype are 0.9071 and 0.8841, respectively, indicating the high recognition success rate for MES by the final classifier, so the final classifier gives satisfactory performance for resolving the triple-class problem.

**Table 2:** The prediction results of the final classifier constructed by RBF-based SVM using a 10-fold cross-validation test on the whole dataset (397 samples).

GBM subtypes	ACC	Precision	Recall	*F*1
CL	–	0.8467	0.8151	0.8306
MES	–	0.9071	0.8841	0.8955
PN	–	0.8019	0.8870	0.8423
Overall	0.8538 ± 0.0280	0.8519 ± 0.0297	0.8621 ± 0.0229	0.8525 ± 0.0292

### Prognostic value determination and establishment of immune signature-based risk score

**Figure 2:**
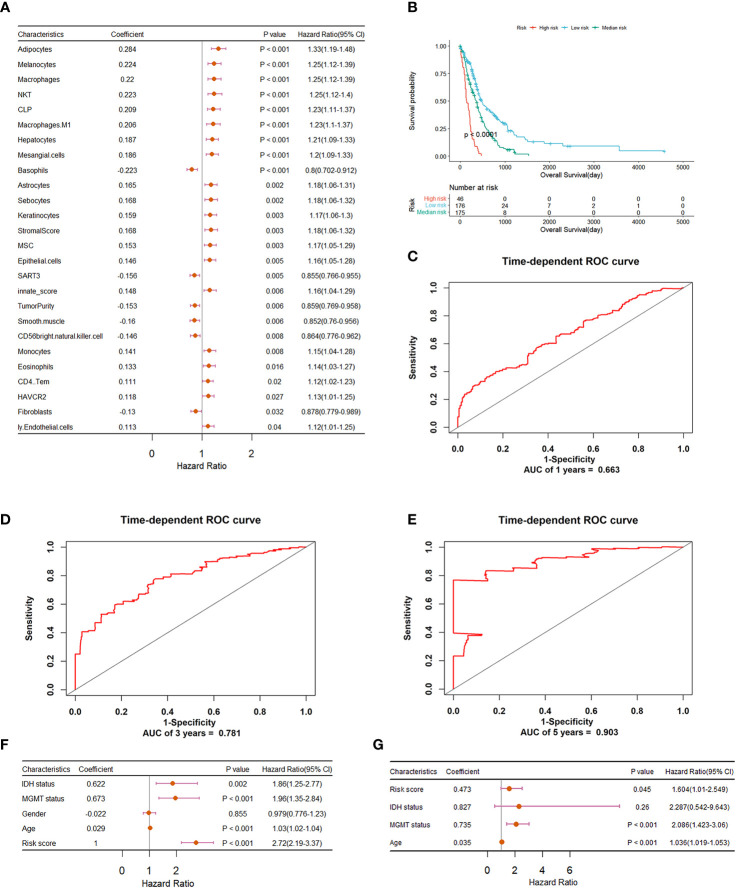
Construction and evaluation of the risk prognostic model based on immune features for GBM patients. **(A)** Forest plot summary of univariate Cox regression analysis of immune features significantly associated with overall survival. **(B)** Kaplan–Meier survival analysis of GBM patients that were divided into high-, medium-, and low-risk groups using a cutoff determined by X-tile. **(C–E)** ROC validation of the prognostic value of the predictive signature for predicting 1-, 3-, and 5-year survival of GBM patients, respectively. **(F)** Forest plot summary of the univariable analysis of IDH status, MGMT status, gender, age, and risk score. **(G)** Forest plot summary of the multivariable analysis of risk score, IDH status, MGMT status, and age. Here, the wildtype and mutant status of IDH and the unmethylated and methylated status of MGMT promoter were both converted to 1 and 0 respectively.

We explored the potential prognostic values of 61 immune characteristics employed in the diagnostic classifier. The prognostic value determination was performed on all GBM samples by univariate Cox regression analysis. The forest plot in **Figure 2A** demonstrates that there are 26 immune signatures that are significantly associated with the OS of GBM. Stepwise multivariate Cox regression analysis was then implemented to further select an optimal combination from these 26 immune signatures. Thus, 13 were identified and used to construct the risk score classifier model. Based on the risk score model, we divided patients into high-, medium-, and low-risk groups using cutoff risk scores determined by X-tile. From the Kaplan–Meier curve analysis in **Figure 2B**, it can be seen that high-risk patients have a shorter survival rate compared to those in the medium- and low-risk groups (*p*< 0.0001). The ROC curves in **Figures 2C–E** indicate that an immune signature-based risk score could sensitively predict OS with AUC values of 0.663, 0.781, and 0.903 for 1-, 3-, and 5-year OS, respectively. In addition, as shown in **Figures 2F, G**, univariate and multivariate Cox regression analyses revealed that the risk score developed by us is independent of MGMT status and age. So these immune signatures could be the prognostic indicators for GBM patients’ OS.

### Differences across GBM subgroups on immune, clinical, and molecular features

**Figure 3:**
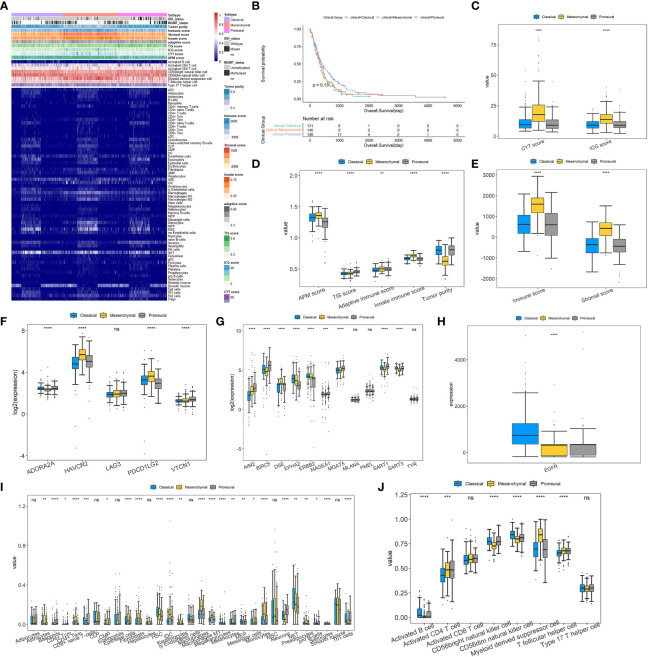
Landscapes of tumor immune microenvironment and clinicopathological characteristics of three GBM subtypes. **(A)** Heatmap depicting the association between GBM subtypes and immune cell infiltration. **(B)** The Kaplan–Meier curve for the OS of 397 GBM patients in three GBM subgroups. **(C–E)** Box plots for exploring the differences of CYT, ICG, APM, TIS, adaptive immune, innate immune, immune and stromal scores, and tumor purity among GBM subtypes. **(F)** Differences in the expressions of T-cell exhaustion markers between the three GBM subtypes. **(G, H)** Differences in the expressions of glioma antigens across the three GBM subtypes. **(I, J)** Different proportions of various immune and stromal cells in the GBM subgroups. For all box plots, the Kruskal–Wallis test was used to determine the significance of differences among GBM subtypes, and *p*-values are shown on the top of each box plot. ^*^ *p*< 0.05; ^**^ *p*< 0.01; ^***^ *p*< 0.001; ^****^ *p*< 0.0001; ns, no significant difference.

Since the immune signatures have been proven to give diagnostic and prognostic values, a differential analysis was performed on them among the three subtypes. Moreover, the clinicopathological characteristics and molecular functions of three subtypes were also proposed. **Figure 3A** displays the heatmap of clinical and immune-related features across three subgroups. It is clear that the proportion of MES patients with IDH mutations is much lower than that of IDH-mutant PN patients. Exactly, there are seven of 131 CL patients, nine of 140 MES patients, and 25 of 126 PN patients with IDH mutations. The previous study has shown that IDH-mutant GBM patients are enriched in the PN subgroup, and those with IDH mutations show a better prognosis than IDH-wildtype cases (53). Moreover, 30 of 131 CL patients, 29 of 140 MES patients, and 29 of 126 PN patients have methylated MGMT promoter, so the percentage of PN patients with methylated MGMT promoter is higher than those of CL and MES; to be more exact, MGMT promoter methylation status has been indicated to be associated with better survival in GBM (54). As shown in **Figure 3B**, the Kaplan–Meier curve analysis reveals that PN patients have a favorable prognosis of OS, which was consistent with previous studies. So, based on the clinical difference between the three subtypes, we can conclude that PN samples exhibit better prognosis than the other two subtypes.

For the nine major immune-associated scores shown in **Figures 3C–E**, compared to CL and PN, MES has been revealed to have higher CYT, ICG, APM, adaptive immune, innate immune, immune, and stromal scores. As seen in **Figure 3F**, except for LAG3, the other four T-cell exhaustion markers are all differentially expressed among three types with *p*-values of<0.0001, although FC values did not exceed 2. Among them, HAVCR2 and PDCD1LG2 are significantly upregulated, while ADORA2A is downregulated in the MES subtype. The differences in antigens are displayed in **Figures 3G, H** which indicate that there is only one antigen, DSE, that is overexpressed within the MES subset. Inversely, several antigens are significantly overexpressed in CL and PN subsets, containing AIM2, BIRC5, MAGEA1, MGAT5, and SART1 in the PN subset and EPHA2, ERBB2, and EGFR in the CL subset.

Given the vital role of TIME, the associations between GBM subtypes and immune infiltration were explored. **Figures 3I, J** illustrate that most immune cells and stromal cells used for constructing the final classification model have remarkable differences in proportions across three GBM subtypes. Compared with CL and PN, MES has a significant higher percentage of myeloid-derived suppressor cells (MDSC), T follicular helper cells, astrocytes, fibroblasts, and macrophages but a much lower proportion of CD56bright natural killer cells and CD56dim natural killer cells. It is consistent with the research by Maria et al. (6) that GBM gives high levels of intratumor heterogeneity in immune infiltration and MES has the highest proportion of macrophage and lymphocyte infiltration. Furthermore, it has been reported that MDSCs can inhibit the immune response by inhibiting the antitumor activity of cytotoxic T cells, NK cells, macrophages, and dendritic cells while inducing Tregs and Bregs, and increased circulating MDSCs are relevant with poor prognosis and survival in GBM patients (55, 56). The accumulation of MDSCs may induce immunosuppressive mechanisms and lead to GBM progression (57). Astrocytes, the main component of the GBM microenvironment, actively participate in the development of this disease through modulation of, for example, migration, invasion, angiogenesis, and therapeutic resistance (58, 59). As the major components of the cancer stroma in solid organ tumors, fibroblasts are called cancer-associated fibroblasts (CAFs), and a variety of biologically active substances, including proteins of the extracellular matrix and growth factors produced by them, may promote glioma cell growth (60). Moreover, CAFs may have a significant role in the invasiveness of GBM and may cause resistance to traditional therapy (9). The findings of Di Ianni et al. reveal that macrophages play a vital role in GBM relapse in a significantly immunosuppressive context (61). The previous study has shown that tumor-associated macrophages comprise 36.39% of the tumor tissue cells and have a subtype-specific role in GBM (62). According to the above findings, we can speculate that MES may be more immunosuppressive than CL and PN subtypes.

**Figure 4:**
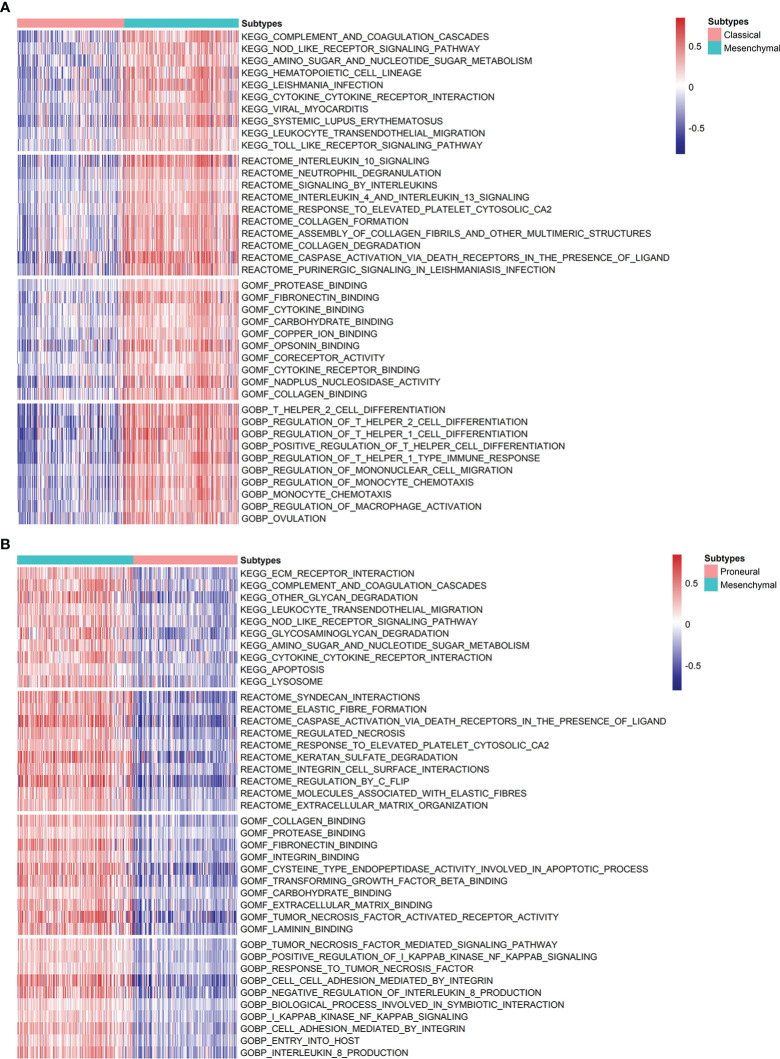
Analysis of the differences in the enrichment scores of KEGG pathways, Reactome categories, and GO terms demonstrated by GSVA among GBM subtypes. **(A)** Heatmap describing the top 10 significantly differential signatures, including KEGG pathways, Reactome, and GO terms between MES and CL. **(B)** Variants in KEGG pathways, Reactome categories, and GO terms between MES and PN.

Lastly, the dysregulated biological functions and signaling pathways were investigated by GSVA analysis. Overall, there is no obvious difference between CL and PN, but MES gives a significant difference from CL and PN, respectively, as shown in **Figures 4A, B**. For KEGG pathways, the immune-associated pathways are highly enriched in MES, such as complement and coagulation cascades, the NOD-like receptor signaling pathway, leukocyte transendothelial migration, and Toll-like receptor signaling pathway. Moreover, interleukin 10 signaling, signaling by interleukin, interleukin 4, and interleukin 13 signaling are also enriched in MES, suggesting a significant association with immune and inflammation responses in MES patients. Except for immune-related Reactome terms, MES is also involved in cancer-associated Reactome terms, such as regulated necrosis, integrin cell surface interactions, regulation by c-FLIP, and extracellular matrix organization, which indicates that patients within MES may be inclined to apoptosis and migration. In addition, immune-related biological processes, such as T helper 2 cell differentiation, regulation of T helper 1 cell differentiation, regulation of T helper 1 type immune response, and regulation of macrophage activation, and cancer-associated biological processes of I-kappaB kinase/NF-kappaB signaling, as well as cell–cell adhesion mediated by integrin, are also enriched in the MES subset. For molecular function, MES is correlated with those that are associated with cancer cell migration and immune response, such as fibronectin binding, cytokine binding, integrin binding, and cytokine receptor binding. Based on the above results, the MES subtype is probably more proinflammatory and invasive than others.

### Analysis of the status of exhausted CD8+T cells and anti-PD-1 immunotherapy response prediction

**Figure 5:**
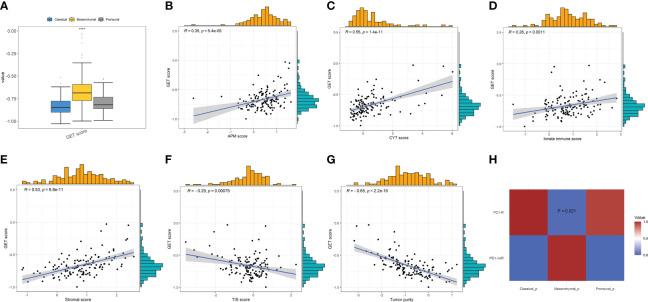
**(A)** Comparisons of GET scores among the three GBM subtypes. **(B–G)** The correlations between GET score and the APM, CYT, innate immune, stromal, TIS scores, and tumor purity, respectively, in MES patients by Pearson’s correlation analysis. **(H)** Immunotherapy response prediction by SubMap analysis indicates a significant difference in anti-PD1 therapy response across the GBM subtypes.

Exhausted CD8+T cells are uniquely marked by distinct PD-1 upregulation. A GET signature was constructed, including 21 genes showing significantly positive correlations with PD-1 levels. They are CD27, SIRPG, CXCR6, ICOS, RUNX2, TNFRSF9, CD70, CD200R1, CD80, TNS3, KIR2DL4, ZBED2, TNIP3, SEMA4A, BATF, TIGIT, VDR, CTLA4, LAG3, KLRB1, and TNFRSF18, respectively. Among them, some are closely correlated to T-cell dysfunction and coregulation, such as CD27, ICOS, RUNX2, CTLA4, etc. Thus, the GET score of each patient was established using the ssGSEA method. We compared the GET score distributions of samples in three subtypes to quantitatively illustrate the status of exhausted CD8+T cells in each subtype. **Figure 5A** shows that there are significant differences in average GET scores across CL, MES, and PN. In particular, GET scores of patients within the MES subtype are remarkably higher than others, suggesting that patients with the MES subtype may have a positive effect on the immunotherapy response. Thus, relationships between the GET scores of MES samples and APM score, CYT score, innate immune score, stromal score, TIS score, and tumor purity were further estimated (**Figures 5B–G**). We can see that the GET score yields positive correlations with the APM score, CYT score, innate immune score, and stromal score, but negative correlations with the TIS score and tumor purity. It has been pointed out that the APM score is associated with inflammatory activities, whereas cytolytic activity represented by the CYT score is relevant to T-cell exhaustion to make the inflamed TME (18, 49). Therefore, we could draw the conclusion that there are probable coordinate interactions among T-cell exhaustion, antigen presentation, and cytolytic activity that could shape the high inflammation in the TME of MES.

The anti-PD-1 immunotherapy response prediction was then conducted. Based on the SubMap analysis, MES patients share a higher similarity with the expression profile of patients that are responsive to PD-1 inhibitor treatment (*p* = 0.021) in **Figure 5H**, so patients belonging to the MES subtype may have significantly better anti-PD-1 responses than others of CL and PN. In fact, previous studies have indicated that patients with high CYT/ICG/APM scores may respond particularly well to immunotherapy, such as immune checkpoint blockade (18, 63). These findings further demonstrate that MES patients with higher CYT/ICG/APM scores may have a better potential response to anti-PD-1/L1 immunotherapy.

### Drug sensitivity prediction and determination of therapeutic drugs

**Figure 6:**
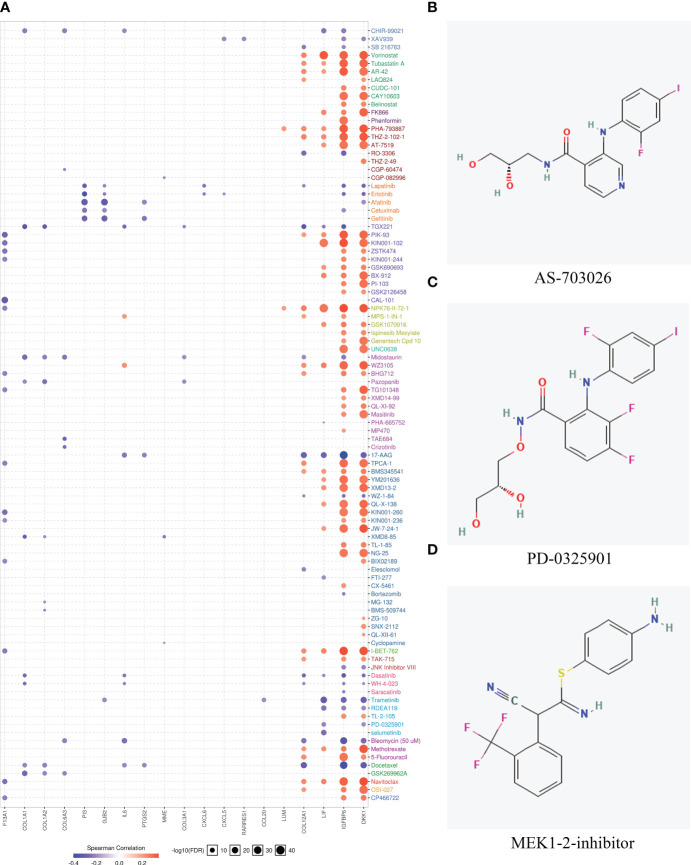
Drug sensitivity evaluation based on hub genes and potential drug prediction for patients within the MES subtype. **(A)** The bubble plot showed the correlation between the mRNA expression of genes that were upregulated in the mesenchymal subtype and GDSC drug sensitives. **(B–D)** Structures of the three most significant bioactive chemicals sharing common MOA of MEK inhibitor by CMap analysis.

Genes with expressions influencing clinical response to drug treatments may be potential biomarkers for drug screening expressions. Since patients within the MES subtype show high immune heterogeneity compared to CL and PN, the potential drugs for MES were finally explored. Firstly, we screened the upregulated genes of MES and calculated the correlations between upregulated gene expressions and drug sensitivity-associated expression profiles from the GSCA database. The results in **Figure 6A** indicate that the overexpression of most genes is negatively correlated with the IC_50_ values of most drugs in GDSC. Among them, 17-AAG, docetaxel, and erlotinib are the top three most negatively related drugs, and all of them are already used in clinical treatments. It has been confirmed that 17-AAG is able to inhibit the growth of both human glioma cell lines and glioma stem cells *in vitro* and could cross the BBB because of its highly lipophilic nature, suggesting that GBM patients may benefit from 17-AAG either as a single agent or in combination with other drugs (64–66). As a semisynthetic taxane, docetaxel is a class of anticancer agents that bind to and stabilize mocrotubules, thereby causing cell-cycle arrest and apoptosis (67). For GBM, although docetaxel could inhibit brain tumor growth following local injection in a mouse brain tumor model, it is unable to accumulate in the brain at adequate concentrations required for tumor regression due to the blood–brain barrier (BBB) and consequently has not been applied to treat brain tumor (68). However, the work by Gajbhiye et al. has revealed that docetaxel-loaded polysorbate 80-anchored dendritic nanoconjugate has the potential to cross BBB significantly and can deliver a higher amount of drug to the brain for a higher therapeutic outcome (69). Erlotinib is an inhibitor of EGFR and has been approved to treat non-small cell lung and pancreatic cancers and was shown to exert multifarious antineoplastic effects in glioblastoma in preclinical studies (70, 71). Erlotinib could be co-delivered with curcumin *via* nanomicelles and show anti-GBM activity in the U87 cell line (72).

Moreover, the CMap database was used to predict potential drugs for patients belonging to the MES subtype. CMap mode of action (MoA) analysis disclosed a total of 43 mechanisms of action in the top 50 compounds. It is noted that three compounds including AS-703026, PD-0325901, and MEK1-2-inhibitor share MEK inhibitors and target two common genes of MAP2K1 and MAP2K2. The chemical structures of the three compounds are shown in **Figures 6B–D**, and the detailed information about them derived by CMap analysis is listed in **Table 3**. AS703206 is a novel, selective, and orally bioactive MEK1/2 inhibitor that has potent cytotoxicity on tumor cells for the majority of patients with relapsed and refractory multiple myeloma (73). Moreover, AS703026 could also effectively inhibit the growth of colorectal tumor cell lines *in vitro* and *in vivo* (74). As an ATP noncompetitive selective inhibitor of MEK1/2, PD-0325901 displays significant antitumor effects in melanoma, head and neck, and BRAF-mutated papillary thyroid cancer (75, 76). It has been reported that PD-0325901 could block the dispersal of GBM by inhibiting the MAPK/EPK pathway, so it is a promising candidate drug as a treatment for intracranial malignancies (77, 78).

**Table 3:** Three bioactive compounds with one common action mode by CMap analysis.

Name	Score	Description	Target	MOA
AS-703026	−99.58	MEK inhibitor	MAP2K1, MAP2K2	MEK inhibitor
PD-0325901	−98.73	MEK inhibitor	MAP2K1, MAP2K2	MEK inhibitor, MAP kinase inhibitor, and protein kinase inhibitor
MEK1-2 inhibitor	−98.03	MEK inhibitor	MAP2K1, MAP2K2	MEK inhibitor

## Discussion

GBM is a common intracranial tumor with a high degree of malignancy, fast growth, a high frequency of recurrence, and few long-term survivors (79). At present, the standard of care (SOC) management of GBM is based on the maximum safe surgical removal, radiotherapy, and chemotherapy with temozolomide. However, patients with GBM still have a poor prognosis, and SOC will lead to irreversible toxicity, such as neurological deficits due to surgery, neurocognitive impairments with radiotherapy, and systemic toxicity (80, 81). It has been confirmed that immunotherapy is highly effective in inhibiting cancer regression and improving patient quality of life (16). However, GBMs exhibit a high degree of inter- and intratumor heterogeneity. Different GBM subtypes exhibit different characteristics of immune landscapes. Hence, a complete understanding of the immune landscape of patients within different GBM subgroups may be beneficial for personalized therapeutic strategies. However, no comprehensive analysis of the immune landscapes of GBM subtypes by integrating various immune characteristics has been reported.

In the present study, we tried to collect a variety of immune signatures to describe the immune landscape of GBM. The comprehensive immune landscape, including 109 immune characteristics, was established for 397 GBM samples compiled from different datasets. Among the 109 immune signatures, 64 immune and stromal cell infiltrations can systematically represent the TME of GBM. The various immune-related scores were calculated. For example, the CYT score assesses the cytotoxic T-cell infiltration, the TIS score quantifies T-cell infiltration levels, the APM score estimates the immunogenicity of tumor cells, the ICG score figures immune checkpoint genes expression, the innate and adaptive immune scores evaluate the levels of innate and adaptive immune activity, and the immune and stromal score represents the overall level of immune and stromal cells. Moreover, T-cell exhaustion markers (11 genes) and 17 genes of glioma antigens were also achieved for the integrative immunogenomic analysis.

The accurate recognition of GBM subtypes is essential for the precise diagnosis and correct treatments of GBM. Here, ML methods were proposed to construct an optimal immune feature-based classification model for simultaneously distinguishing three GBM subtypes of CL, MES, and PN, respectively. Usually, when there are too many features in an ML model, there may be some that are redundant or irrelevant, which probably reduces the classification performance of the ML model. Feature selection could obtain a high-quality feature subset by removing irrelevant and redundant data (82). A high-quality feature subset could improve learning accuracy, reduce computational overload, and simplify learning results (83); hence, feature selection is the key step in model construction. The diagnostic values of these collected immune signatures were investigated using SVM-RFE, and 61 optimal immune signatures were selected. Seven different ML methods were compared, and the RBF-based SVM model gives the best performance, with an overall prediction accuracy of 85.38% in the final model by 10-fold cross-validation. For the MES subtype, the recognition precision is as high as 0.9071, indicating the high diagnostic value of these immune signatures. In addition, there have been existing ML models for GBM subtype classification based on different data; for example, Munquad et al. (84) utilized transcriptome and methylome data to construct classifiers through several ML algorithms, and the best model presents an accuracy of 87.5% on the testing data and 94.48% on external data. Macyszyn et al. (85) employed magnetic resonance imaging and the ML method to identify molecular subtypes in GBM with a prediction accuracy of 76%. Zhang et al. (86) constructed an SVM classifier for dividing GBM into seven subtypes based on DNA methylation status with an overall accuracy of 85.2% on the independent test dataset. It can be observed that our classifier based on immune signatures yields comparable performance with the existing ML models based on other feature information. However, so far, no other research has been reported combining immune features and ML algorithms for GBM subtypes classification. The model constructed by us could be a promising supplementary tool for the accurate recognition of GBM subtypes.

The clinical relevance of the 61 immune features in GBM was assessed by survival analysis, and 26 of them were found to be correlated with the OS of GBM patients. Therefore, a prognostic signature was constructed for predicting GBM’s OS. The Kaplan–Meier analysis suggests significant differences in survival times among high-, medium-, and low-risk patients. Furthermore, the ROC analysis proved that the prognostic signature could precisely predict long-term survival than short-term survival of GBM patients, with an AUC of 0.903 for a 5-year OS of GBM. In addition, the risk score could be an independent, applicable prognostic indicator of GBM after adjusting for clinical factors including gender, age, IDH, and MGMT status. The 26 immune signatures may be potential prognostic indicators for GBM patients’ OS.

TME of cancers has been known as a crucial aspect for understanding antitumor response and sensitivity to immunotherapy (15). Through analyzing the difference in immune and stromal cell infiltration across three GBM subgroups, it has been found that patients within MES have much higher percentages of MDSC, T follicular helper cell, astrocytes, fibroblasts, and macrophages, which could inhibit immune response and promote invasion, migration, and cancer development. Moreover, the comparisons of enriched pathways among GBM subgroups show that a number of oncogenic and immune-associated pathways are significantly upregulated in MES, such as I-kappaB kinase/NF-kappaB signaling, regulation by c-FLIP, extracellular matrix organization, NOD-like receptor signaling pathway, Toll-like receptor signaling pathway, and leukocyte transendothelial migration. The activation of the I-kappaB kinase/NF-kappaB signaling pathway could not only lead to the induction of target genes associated with apoptosis, cell cycle regulation, cell invasion, and metastatic growth but also regulate cancer-related inflammation, hyperplasia, and neoplasia (87, 88). As a master regulator of death receptor networks, c-FLIP plays a key role in apoptosis, necroptosis, NF-κB activation, and tumorigenesis, and high c-FLIP levels are correlated with a more progressive tumor and critical for inflammation (89, 90). It has been indicated that NOD-like and Toll-like receptors are essential players in the innate immune response to invading pathogens and are linked with human diseases, including infections, cancer, and autoimmune and inflammatory diseases (91). In cancer, NOD-like receptors are initiators of the inflammasome pathway and directly facilitate tumor cell growth and metastasis, then help prevent any antitumor immune response (92). Toll-like receptors can activate NF-κB and promote tumorigenesis and proliferation (93). To sum up, these findings indicate that there is a more immunosuppressive and inflammatory TME in the MES subtype than in CL and PN. Fan et al. (94) have proven that patients with a high risk of glioma are observed to retain a more activated inflammatory state but more suppressive TME, which is consistent with our conclusion that patients within the MES have a poorer prognosis than those within the CL and PN.

As for the differences in immune-associated scores among GBM subgroups, MES is revealed to have higher APM, CYT, and ICG scores but a lower tumor purity. Chen et al. (25) show that the APM signature score could predict an immunosuppressive and onco-inflammatory microenvironment supporting tumor growth and progression. In contrast to other cancers like hepatocellular carcinoma, a high CYT score is associated with higher expressions of immunosuppressive PD1/PDL1 axis in GBM and also relates to worse OS. However, it has been indicated that patients with a high CYT/ICG score may respond particularly well to immunotherapies because GBM patients have a complex microenvironment with increased ICG expression and protumoral immune cell infiltration (63). Additionally, tumor purity is observed to significantly correlate with the reduced survival time in GBM (95). Thus, MES patients might have a poor prognosis and be more sensitive to checkpoint-related immunotherapy.

Meanwhile, we established a GET score to investigate the dysfunctional immune state among GBM subtypes. MES yields a higher GET score and gives more significant correlations between the GET score and the APM score, CYT score, innate immune score, stromal score, TIS score, and tumor purity, respectively, which proves that T-cell exhaustion, antigen presentation, and cytolytic activity may corporately shape the complex and inflamed TME in MES. Since the GET score is correlated with better clinical benefit for ICI agents (50), MES may have a positive effect on the immunotherapy response, which is further confirmed by SubMap analysis, which shows that MES patients are more likely to benefit from anti-PD1 treatment.

Considering the high immune heterogeneity of MES, disclosing the potential drugs may improve the medical therapy and prognosis of MES patients. We finally examined the drug sensitivity of several anticancer drugs based on upregulated genes in the MES subgroup. The GSCA analysis demonstrates that drugs such as 17-AAG, docetaxel, and erlotinib exert antitumor activity with corresponding genes. These drugs have been approved to treat various cancers after extensive research. As an analog of geldanamycin, 17-AAG has been widely investigated in the preclinical and clinical research as a single agent or in combination/noncombination with other anticancer agents for various cancers, such as breast cancer, ovarian cancer, prostate cancer, glioblastoma, etc. (96). Docetaxel has shown profound benefits in the treatment of diverse cancers (breast, head, neck, lung, and prostate cancer), and erlotinib have been approved to treat non-small cell lung and pancreatic cancers (70). Moreover, used CMap was used to disclose potential drugs for MES patients. Three compounds (AS-703026, PD-0325901, and MEK1-2-inhibitor) were identified that share MEK inhibitors and target two common genes (MAP2K1, MAP2K2). However, the practical applicability of those drugs would be experimentally confirmed in future studies.

In summary, this study comprehensively analyzed the immune landscape in GBM and found that the MES subtype could be considered an immunosuppressive, proinflammatory, and invasive subtype. However, MES has better responses to anti-PD-1/L1 immunotherapy. This research could provide a theoretical basis for identifying GBM subtypes by the immune signatures, followed by the development of more effective, targeted clinical treatment strategies, and finally, achieving precision medicine.

## Data availability statement

The original contributions presented in the study are included in the article/**Supplementary Material**. Further inquiries can be directed to the corresponding author.

## Author contributions

HL proposed the methodology, collected and processed all data, conducted the statistical analysis, and wrote the original draft. JH performed a formal analysis. ML, KL, and XP completed validation, writing reviews, and editing. YG conceived and designed the project, supervised the project, and wrote and edited the manuscript. All authors discussed the results and contributed to the final manuscript.

## Funding

This research was financially supported by the Foundation from the Science and Technology Department of Sichuan Province (2020JDJQ0017, 2022YFG0185) and the Sichuan International Science and Technology Innovation Cooperation Project (2021YFH0140).

## Conflict of interest

The authors declare that the research was conducted in the absence of any commercial or financial relationships that could be construed as a potential conflict of interest.

The handling editor LZ declared a shared parent affiliation with the authors HL, JH, ML, KL, XP, YG at the time of the review

## Publisher’s note

All claims expressed in this article are solely those of the authors and do not necessarily represent those of their affiliated organizations, or those of the publisher, the editors and the reviewers. Any product that may be evaluated in this article, or claim that may be made by its manufacturer, is not guaranteed or endorsed by the publisher.
